# Protocol for a phase III wait-listed cluster randomised controlled trial of an intervention for mental well-being through enhancing mental health literacy and improving work friendliness in Hong Kong

**DOI:** 10.1186/s13063-019-3748-y

**Published:** 2019-12-04

**Authors:** Lawrence T. Lam, Prudence Wong, Mary K. Lam

**Affiliations:** 1 Tung Wah College, 31 Wyloe Road, Homintin, Hong Kong SAR, Kowloon China; 2 Faculty of Health, University of Technology Sydney, 235 Jones Street, Ultimo, Sydney, Australia; 3 Mental Health Association Hong Kong, Hong Kong SAR, China

**Keywords:** Mental health literacy, Work stress, Burnout, Health-related quality of life, Mental well-being, Workplace intervention, Psychoeducation, Work environment, Mental health promotion, Mental public health, e-Health

## Abstract

**Background:**

Mental health has long been recognised as a major global health issue. Some work-related characteristics have been identified to be associated with common mental health problems, and thus the workplace is an important venue for the prevention of mental health problems and promoting mental wellness. Burnout is one of the important aspects of workplace organisational stressors and, in recent years, the lack of mental health literacy has also been identified as a fundamental issue. Studies have demonstrated that an improvement in mental health literacy is an effective measure for enhancing mental well-being. It would be prudent to combine an organisation-directed component and the enhancement of mental health literacy in an intervention programme. This trial will examine the novel approach of an intervention aiming to provide an evidence-based prevention programme.

**Methods:**

This study utilised a wait-listed cluster randomised control trial design. Using branch offices as the primary sampling units, employees from three large companies in different industries will be recruited. Upon enrolment and after the baseline assessment of the outcome measures, participants nested in the branch offices will be allocated to the intervention or wait-listed arms. The intervention programme comprises of two main elements: an organisation-directed component and individual-directed psychoeducation training. This intervention will be delivered by a senior social worker well-versed in workplace issues over a period of 3 months. The trial will determine whether an integrated workplace mental health literacy and well-being programme is effective in increasing the mental health literacy scores and reducing burnout and stress scores, as measured by standardised and validated scales.

**Discussion:**

If the trial results are in line with the hypothesis that supports the efficacy of the intervention programme, this will provide an evidence-based approach for an effective workplace mental well-being intervention programme that could not only enhance the understanding of mental health issues, but also reduce work-related burnout and stress as well as increase workers’ quality of life.

**Trial registration:**

Australian New Zealand Clinical Trials Registry (ANZCTR), ACTRN12619000464167. Registered prospectively on 20 March 2019.

## Background

Mental health has long been identified as a major global health issue. Mental and substance disorders are the leading cause of years lost due to disability (YLD), accounting for about 21.2% of the global burden of diseases [1]. A recent study with more precise calculations has revised the burden to 32.4%, rendering mental illnesses the top cause of YLD [2]. In Hong Kong, while information on the overall mental health status has been scarce, results obtained from the Hong Kong Mental Morbidity Survey (HKMMS) suggested that common mental disorders were the most prevalent [3]. The point prevalence of mixed anxiety and depression disorders was estimated to be 13.3% in the adult population aged between 16 and 75 years [3]. Among these, only slightly more than a quarter (26%) sought help from mental health services and fewer than 10% consulted a general practitioner [3]. The latest Mental Health Review Report revealed that, based on the international prevalence of mental disorders and extrapolation, there could be as many as 1.1–1.8 million individuals experiencing a mental disorder in the adult population [4].

It has been estimated that, on average, full-time workers in the Organisation for Economic Co-operation and Development (OECD) countries spent about 38% of time in a normal day working (www.oecdbetterlifeindex.org/topics/work-life-balance/). While work is an essential part of our life and brings many benefits, some work-related characteristics have been identified to be associated with common mental health problems [5]. In a recent large-scale study on the mental and physical health of a working population, Rose et al. [6] found that work-related stress and fatigue were associated with mental health problems such as depression. While there has not been a wealth of studies on the mental health status in the workplace in Hong Kong, some other studies on specific topics have shed light on the issue of poor mental health and its effect on working individuals. A psychological autopsy conducted by Law et al. [7] found that, in a comparative study of 63 employees who committed suicide and 112 controls, psychiatric illnesses played an important mediating role in the association between chronic work-related stress and suicide. This suggested a potentially causal pathway between work-related stresses, mental ill health, and, subsequently, suicide [7]. In another recent study on suicide ideation and attempts among nurses in Hong Kong, it was found that about 15% had contemplated suicide and about 3% actually attempted suicide once or more in the year prior to the survey [8].

Mental health in the workplace has long been an important health concern internationally. The workplace has also been identified as an important venue for the prevention of mental health problems and for promoting mental wellness [9]. This, in fact, is also a main focus of the World Health Organization (WHO) comprehensive mental health action plan 2013–2020. The plan stated the paramount importance of prevention of and early intervention in mental health problems in the community, including the workplace [10]. Hence, improving mental wellness of the working population should be considered an important primary preventive strategy. Within the working context, this can be achieved through an early intervention programme designed specifically for employees in the workplace.

Many workplace intervention programmes have been developed in the past, with the majority mainly focused on one important aspect of work-related stressors, namely burnout [10]. A review study on the effectiveness of various intervention programmes in reducing burnout found that there were two main types of intervention programmes: person-directed and organisation-directed programmes [11]. Results of the review indicated that person-directed intervention programmes tended to be effective for a short term of 6 months or less, whereas intervention programmes directing at the organisation level achieved a longer-term effect of 12 months or beyond. As a result, it was suggested that any workplace intervention programmes should be designed with dual foci directed at both the organisation and individuals.

While acknowledging that burnout is an important aspect of workplace stress, it is only one of many pieces of the puzzle. In addressing the issue of mental health problems and to advance mental wellness in the community, attention has been drawn to a more fundamental issue, namely the lack of mental health literacy (MHL). MHL is a concept developed by Jorm et al. [12] about two decades ago. MHL has been defined as “knowledge and beliefs about mental disorders which aid their recognition, management or prevention” [12]. Since then the concept as well as the assessment methodology have been widely adopted. It has been argued that lack of understanding and awareness of mental health problems, compounded with self-and-other stigmatisation of these problems, were major barriers in help-seeking for people in need [13]. The reluctance and inaction in help-seeking resulted in the individual not receiving the appropriate and timely mental health assistance and, in turn, might exacerbate the condition [13]. In order to tackle the root of mental health problems, improving MHL was suggested to be an effective strategy for enhancing self-help-seeking behaviour or assisting others to seek help [13]. This in turn, might result in early diagnosis and treatment, and thus lighten the burden of mental illnesses in the population.

To determine the effectiveness of the approach in addressing the issue, a systematic review and meta-analytical study on the effect of intervention programmes, mainly on help-seeking behaviour, was conducted [14]. The results of the study revealed that interventions on enhancing mental health literacy were shown to be effective in increasing help-seeking attitude with an effect size ranging from 0.12 to 0.53 [14], although the effects on the actual help-seeking behaviour were not certain. On the other hand, a study on the relationship between mental health literacy and depression among young people indicated a significant association between the two. Young people who had experienced moderate to severe levels of depression were more likely to have an inadequate level of mental health literacy (OR = 1.52, 95% CI = 1.01–2.31) [15]. These results suggested that intervention programmes aiming to enhance the mental health literacy of individuals could have a positive effect on their actual mental health status, probably through an increased awareness of the problem, and also strengthened attitudes towards seeking appropriate help.

Given the aforementioned evidence from studies on burnout, it would be prudent to consider combining an organisation-directed component and the enhancement of mental health literacy in an intervention programme. In doing so, the creation of a positive environment can lead to greater efficacy in reduction of mental health problems and enhancement of mental wellness. Such an approach has not received much attention in the area of early intervention and prevention of mental health problems in the workplace. The study team has developed a novel approach to intervention taking into consideration the influential factors in workplace mental well-being. The proposed trial aims to submit this intervention to a proper scientific investigation for proven evidence on its efficacy.

The trial protocol reported here adheres to the Standard Protocol Items: Recommendations for Interventional Trials (SPIRIT) Checklist (see Additional file 1).

## Objectives

### Primary objective

The primary aim of the proposed study is to determine whether a workplace mental well-being intervention programme that responds to organisation and individual mental health needs, based on the dual approach of an organisation environment scan and evidence-based psychoeducation training in mental health literacy, would increase MHL.

### Secondary objectives

The study further aimed to investigate whether the intervention:

- Reduces work-related burnout in workers
- Increases the mental health well-being, particularly in the reduction of stress, of workers
- Increases the general health-related quality of life of workers

## Trial design

**Fig. 1:**
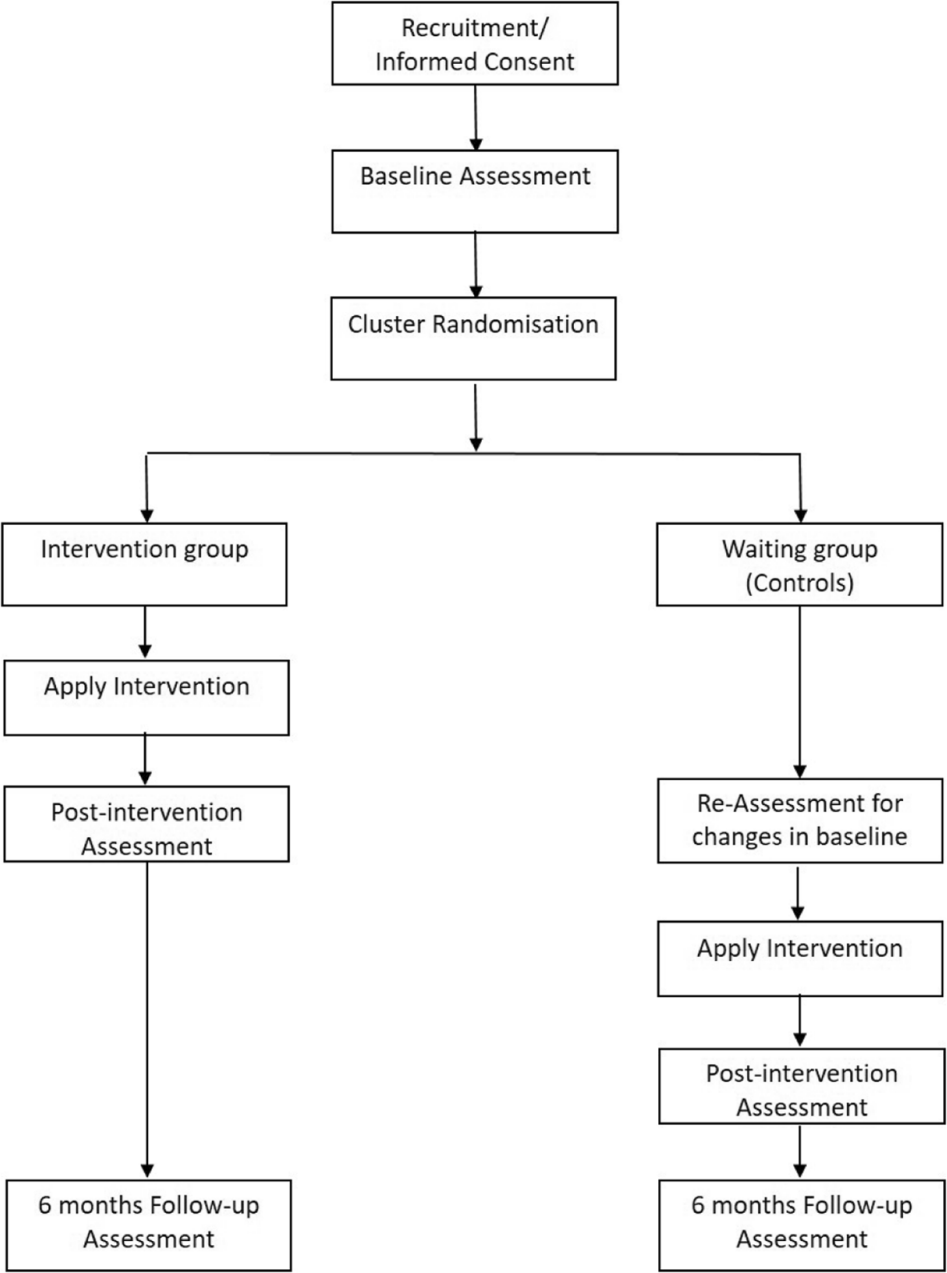
Study flow chart

The study will use a phase III wait-listed cluster randomised control trial (RCT) design with different work sites or branch offices as the primary units of randomisation (refer to Fig. 1).

## Methods: participants, interventions and outcomes

### Study setting

The study will focus on specific industries with a considerable level of work-related stress, such as the servicing and hospitality industries. Employees in these industries may have a large volume of customer or client interaction, or the job nature may carry heavy personal and/or interpersonal responsibilities. Currently, three large companies in Hong Kong have indicated willingness to be involved in the study. These include a company providing travel services, a real-estate company, and a property management company. All three companies have signed an agreement to willingly participate in the project.

### Eligibility criteria

#### Sites

Managers of the participating sites are willing to commit to the project and allow the study to be conducted at their branch offices.

#### Participants

All staff members working at the selected sites are eligible to participate in the study. However, participants have to indicate their willingness to participate in the study and to comply with the requirements of the study by signing the consent form.

### Exclusion criteria

#### Sites

Managers of any sites who are not willing to comply with the requirements of the study and not willing to allow the project team to conduct the workplace environment scan in the branch office will be excluded. Participants who are receiving training on workplace mental health will not be included in the study.

#### Participants

Any staff member at any site who has already received a similar psychoeducation training programme or has been involved in any similar intervention programme before will be excluded from the current intervention programme.

### Intervention

#### Intervention to be tested

The intervention programme comprises two main elements: an *organisation-directed* component and *individual-directed* psychoeducation training.

For the organisation-directed component, a workplace environment scan, using a standard protocol and the Moos Work Environment Scale (WES) [16], will be conducted by a senior social worker with expertise in workplace issues. The scan will take place at the commencement of the intervention programme prior to the individual-directed component. Different dimensions of the work environment, including workload (work pressure and task requirements), personal growth (professional growth, support, achievement value, and growth), conflict, and management relationships, will be assessed to gain a better understanding of the workplace issues at different sites. Information will be gathered via a staff survey and face-to-face interviews. De-identified and aggregated information collected from the assessment will be provided to the management of each participating site with professional interpretation of the findings. Possible strategies to resolve the identified issues will also be offered. Site management is encouraged to make use of this information to improve the work environment during the intervention and the follow-up period. Each site is encouraged to document changes in organisational practices and to, quantitatively and qualitatively, record any changes in the workplace in terms of morale, work performance, productivity, sick leave, and resignations. Information on these changes will be collated at the end of the project through interviews with site managers.

For the individual-directed component, participating sites will adopt the well-studied and evidence-based Workplace Mental Health First Aid (MHFA) training programme [17] with the inclusion of a module on stress reduction and burnout prevention. Based on the e-Health and e-Learning approach, this is blended training consisting of a series of self-paced online e-Learning modules. Participants will have access to these online modules to complete each at their own convenience. Upon completion of the online modules, a face-to-face group session lasting for about half a day will be conducted. The aim of the group session is to provide an opportunity for participants to clarify any queries interactively with the programme trainer and to gain hands-on experience through communication skills practices.

Pilot testing involved a small-scale pilot study conducted by the team on a mental health education and training programme in a sample of high school teachers and nurses in China. This project was set up as a joint initiative between the Australian and Chinese governments through the Australia China Council (ACC) of the Department of Foreign Affairs and Trades, Australian Government (http://dfat.gov.au/people-to-people/foundations-councils-institutes/australia-china-council/grants/grantees/Pages/mental-health-education-for-education-and-health-professionals-in-china.aspx). Although the focus of this project was not on workplace mental health, valuable lessons were learned in terms of cooperation between partners as well as the responses of young professionals towards a mental health education programme. These will inform the development of the current proposed study.

#### Pre-randomisation process

An operational agreement between the project and the site management will be established with the manager signing the consent form. Upon recruitment of participants, a briefing session will be conducted by a qualified senior social worker, trained in workplace psychosocial issues, with the manager of the site and the workers separately prior to the randomisation. The aim of the briefing is to provide details on the study and to obtain informed consent before the baseline assessment is conducted on the workers. During the session, the details and requirements of the study will be explained, and questions and concerns raised will be answered. This will be followed by administration of the baseline assessment (T0).

#### Intervention arm

Consenting participants nested in the sites that are randomised to the intervention arm will receive the intervention immediately (Fig. 1). The intervention will commence with a workplace environment scan. Results of the scan will be fed back to the manager of the site during a professional consultation session. The psychoeducation training programme will be implemented with all willing participants enrolled into the online modules and commencing the MHFA programme. Upon completion of the online modules by all participants at each site, a face-to-face session will be conducted on site at the workplace. At the end of the intervention programme, participants will then be re-assessed on the outcome measures (T1). The sites and participants in the intervention arm will be followed for another 3 months, with re-assessment conducted post follow-up (T2).

#### Control group

Consenting participants randomised to the wait-listed control group will also complete the baseline data collection measures, as those in the intervention group, but will not have access to the training programme until participants in the intervention arm have completed the programme with the re-assessment of the outcome measures. It is anticipated that the intervention programme will be implemented in the control group within 3–4 months after the commencement of the programme in the intervention arm. The same procedures will be applied to the controls as in the intervention arm, with re-assessment of outcome measures, immediately prior to the intervention (T1), at the end of the intervention (T2), as well as at the follow-up re-assessment (T3).

#### Adherence to intervention

Intervention adherence will be monitored weekly via record checking by the Research Assistant. Upon completion of one unit of online training, the participant will be informed automatically by the online platform to commence the next unit of the programme. For participants who are not progressing through the programme as planned, a short reminder message will be sent via common instant messaging applications, such as WhatsApp or WeChat. Should the participants encounter any problems or technical issues with the online programme, assistance will be provided by the project team through a hotline.

### Outcomes

#### Primary outcome measure

The primary outcome measure is the MHL of workers. MHL will be assessed using the Australian National Mental Health Literacy and Stigma Survey designed by Jorm et al. [12]. The instrument has been validated and widely used in many studies and different countries. As part of the international Mental Health First Aid collaboration, the MHAHK has permission from the Australian headquarters to translate and use the survey freely. The Chinese version of the instrument has been validated by the Mental Health Association Hong Kong (MHAHK). It is hypothesised that the intervention programme is efficacious in increasing the level of MHL of workers. A significant increase in the MHL score in the intervention group in comparison to the control group at the end of the intervention period will be able to demonstrate the efficacy of the intervention.

#### Secondary outcome measures

Secondary outcomes include burnout, stress, and health-related quality of life. These will be examined by the following different instruments.

Burnout will be evaluated by the Maslach Burnout Inventory (MBI), which is specifically designed to measure the extent of burnout in adults in the workplace [18]. The instrument consists of three main domains capturing three different aspects of the burnout phenomena in individuals: emotional exhaustion, depersonalisation, and personal accomplishment. Emotional exhaustion measures the feelings that the worker is emotionally exhausted by his/her work. Depersonalisation captures the state that the worker is impersonally responding to recipients of one’s service, care treatment, or instruction. Personal accomplishment measures the level of competence and successful achievement of the worker in providing services [18]. This instrument has been fully validated and has been widely used around the world for many years. It has been translated into many languages. Recent studies on the psychometric properties of the MBI suggested that the three-factor model fitted the data better than other alternative models with high internal consistency for all three subscales, yielding Cronbach’s α values of 0.84, 0.87, and 0.88, respectively [19].

Stress will be assessed by the Anxiety subscale of the Depression, Anxiety, and Stress scale (DASS) [20]. The DASS is a fully validated and commonly used instrument designed for the assessment of stress, depressive symptoms, and anxiety. This scale has good psychometric properties, which include strong reliability and validity for both the English and the Chinese versions [20, 21].

Health-related quality of life (HRQoL) as an indication of general health will be measured by the five-level version of the European Quality of Life-5 dimensions (EQ-5D-5L) [22]. The validity of the EQ-5D-5L has been well demonstrated and widely published (https://euroqol.org/eq-5d-instruments/eq-5d-5l-about/). A Chinese version of the EQ-5D-5L has also been validated and commonly used in many studies [23].

### Participant timeline

#### Sample size

For the sample size, it has been estimated that about 400 workers are required with an estimated effect size of about 0.5 units of a standard deviation difference in the MHL scores between the intervention and control arms [12]. The sample size calculation is based on this assumption with a power of 80% required to detect a true intervention effect at a type I error margin of 5% with an intraclass correlation of about 0.01 for the clusters. It is also assumed that 10% of participants will drop out of the project. Since groups of participants are recruited from different sites with an average size of about 20 workers, a total of 20 sites would be required.

### Recruitment

As aforementioned, three large companies representing different industries and a range of workplace environment have agreed to participate in the study. The study team has connection with the Human Resources Department and Senior Management of these companies. The Human Resources Departments will fully support the recruitment process with encouragement for different site offices to be part of the study. Participants will be recruited from different site offices of these companies through internal advertisement by the Human Resources Departments. Participation in the study is totally voluntary and informed consent will be obtained from participants.

### Consent

Informed consent will be obtained from potential participants during the briefing session conducted by the senior social worker at each site once the site managers have agreed to partake in the study. At the briefing session, information on the study and the intervention programme will be provided to all potential participants verbally and as a written information sheet (Additional file 3). Willing participation in the study will be solicited from potential participants by signing the consent form (Additional file 2). Upon consenting, participants will be asked to provide their mobile number as the unique identifier of the individual so that data collected at different time points of the study could be linked. Potential participants who are unable to attend the briefing session can contact the research team directly prior to consenting. The same consent process will be applied to all sites.

### Allocation

As a cluster randomised trial, the primary unit of randomisation is the site. For random allocation of sites, a full list of all participating office sites with some basic staffing information, such as the number and the ranks and files of staff in the office, will be obtained from the Human Resources Departments of three companies. In accordance with the preliminary information provided by these companies, the average size of site offices is about 20 staff and the deviation is small, thus the only factor for consideration in randomisation is the distribution of the site offices among these three companies. Hence, the randomisation will be stratified in accordance with the number of participating site offices across three companies. Each participating site office will be allocated to an intervention or wait-listed control arm according to a randomisation schedule generated by a central registry. The randomisation will be conducted by a qualified statistician who is blinded to the process of recruitment and the ongoing outcome assessments. The central registry will be responsible for generating the randomisation tables and will provide the information to the field staff right after the briefing session and baseline data collection via instant messaging. Participants and the site manager will be informed of the allocation at the end of the briefing session. The allocation of the randomisation codes with all information on the site office will be managed by the study coordinator. The allocation ratio between the intervention arm and the controls is 1:1.

### Blinding

Allocation is concealed from the study Principal Investigator and the study team at the time of the participants’ inclusion in the trial; however, field staff and the project coordinator collecting and managing the data will not be blinded to participants’ allocation. In no circumstances should the blinding be broken to the study team.

## Methods: data collection, management and analysis

### Data collection methods

Data on the outcome measures will be collected at four time points during the trial in accordance with the schedule depicted in Fig. 1. These are the baseline assessment during enrolment (T0); re-assessment after the completion of the intervention programme for the intervention arm and also for the controls (T1); re-assessment of the controls at the end of the intervention programme and the follow-up assessment of the intervention arm (T2); and follow-up assessment of the controls post intervention (T3).

#### Workplace environment scan

The workplace environment scan will be conducted by the senior social worker using the Moos Work Environment Scale (WES) [16]. Data will be collected during the briefing session through an online survey. Participants from each site will be asked to fill in the survey on site at the end of briefing session. Participants who have consented but miss out on the briefing session can contact the project team. They will be guided to fill in the baseline survey through a telephone call. Incomplete surveys will be identified during the data management process right after baseline data collection. Participants who have not completed the survey will be contacted via instant messaging or emails to encourage a re-attempt of the survey.

#### Outcome measures

**Fig. 2:**
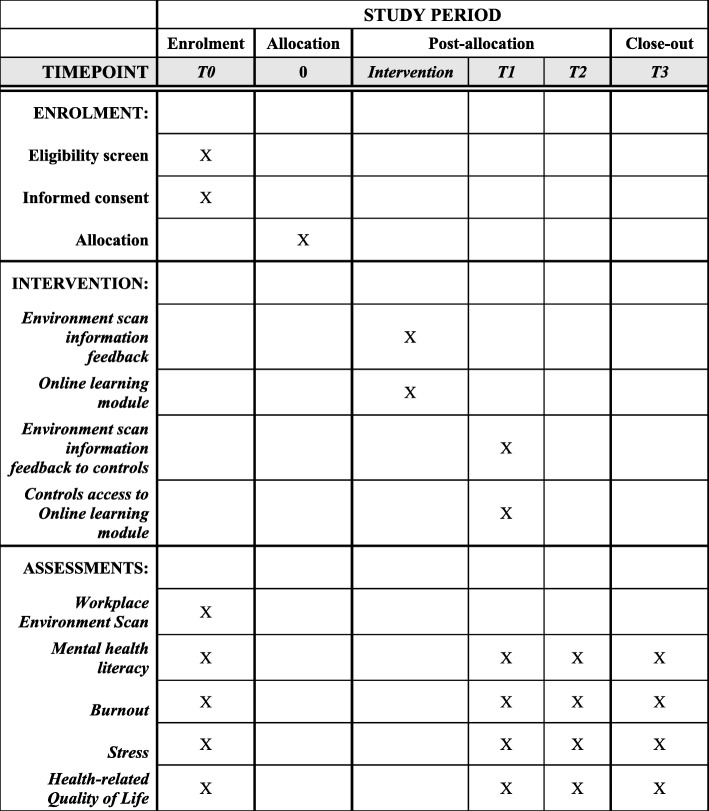
Standard Protocol Items Recommendations for Interventional Trials (SPIRIT) figure. T0 baseline assessment during enrolment, T1 re-assessment after completion of the intervention programme for the intervention arm and also for the controls, T2 re-assessment of the controls at the end of the intervention programme and the follow-up assessment of the intervention arm, T3 follow-up assessment of the controls post intervention

Assessments on the outcome measures will be conducted at different time points in accordance with the time schedule tabulated in Fig. 2. Data on these outcome measures will also be collected using an online survey form. For baseline assessment, data will be collected at the same time as the workplace environment scan. Participants will be asked to fill in both surveys at the same time. All participants will be invited to provide their mobile telephone number on the form before commencing the survey. Since the mobile number will be used as the unique identifier for linking data collected at different time points, it would be important to ensure that they are correctly reported. With the permission of the participants, these mobile numbers will be verified with the records retained by their companies.

### Data management

All hard copies from the study will be kept at the Research Office of Tung Wah College (TWC) located at 31 Wylie Road, Homantin, Kowloon, Hong Kong SAR. All electronic files, including documents generated in the study and the electronic version of the signed consent form, the database developed for the study, and other data sets derived from the database, will be housed and stored in the secure file server designated for research at TWC. All electronic database and any data sets derived from the database will be password protected. The passwords will only be revealed to the key members of the research team and the statistician who will be handling the data for analyses. For further security, personal information provided by participants during enrolment will be kept in a separate database from the other data collected during the study. The only linkage between these databases is via the unique identifier – the mobile phone number provide by the participant. Hard copies of data will be securely stored in a locked file cabinet inside a secured office accessible only by a designated staff member through a security code. All data, both hard and soft copies, will be stored for 7 years.

### Confidentiality

Information on the participants and their responses to the survey will not be revealed to site managers or the Human Resources Departments of the companies. This will ensure that all identifying data can be managed in a confidential manner. Participants are free to withdraw from the study at any time without any request of a reason. Withdrawal of consent will not affect participants’ employment at their current work site (Additional file 2). However, participants will be informed that data that have already been collected may still be used in the analyses, and they will also be advised that it may not be possible to withdraw their data from the study results.

### Statistical methods

#### Statistical analysis plan

The analysis of the primary and secondary outcome measures will be conducted in accordance with the study design as a wait-listed cluster randomised trial and the nature of the variables. An intention-to-treat analysis will be applied to all primary and secondary outcomes. Any missing data will be imputed using the multiple imputation by chained equations approach. Should there be any differences identified between the intervention arm and the wait-list controls by comparisons of the baseline data collected, these variables will be considered potential confounders to be adjusted. A significance level of 5% will be adopted for testing all hypotheses.

The primary outcome measure of the study, namely MHL, will be analysed using a repeated-measures approach with possible adjustments to participants’ and sites’ characteristics. This will involve comparison of the mean change in the MHL scores from baseline to the completion of the intervention programme between the intervention and control groups (i.e. from T0 to T1). Since the study is a cluster randomised trial, the cluster sampling effect will be adjusted for. In order to cater for the sampling technique as well as the repeated measures of the outcome variable, the generalised linear latent and mixed model (GLLAMM) will be applied to test for any group differences.

For secondary outcome measures, the variables are of the same nature as for the MHL, namely continuous variables, and thus the approach of analyses will be the same. For the follow-up data, since all participants will receive the intervention programme, there will not be a distinction between the intervention and control groups and thus all participants will be considered a single group. Hence, the analysis will only focus on the changes in outcome measures across time. For the repeated measures of the outcome variables, a linear mixed-effect model will be applied.

#### Interim analysis

Since the actual trial period will only last for 12 months, and also due to budgetary constraints, the research team has decided not to conduct an interim analysis.

### Methods: monitoring

#### Data monitoring

This is a trial of an intervention that involves mainly a psychoeducation training programme and does not directly involve treatment or management of any participants, and thus no adverse events are anticipated. As such, a data monitoring committee will not be convened. Should any issues arise, they will be dealt with initially by the research team. If the matter is of a more serious nature it will be referred to an independent Project Management Committee (PMC).

#### Study monitoring

Throughout the study period, regular meetings will be held between the Principal Investigator, key members of the research team, and the field staff for monitoring the progress of the study. During these meetings, the project coordinator will provide regular reports to the research team on recruitment, data collection, and any issues arising during these processes. Any perceived/identified irregularities will be referred to the PMC for further discussions and decision. Any deviation of or modification to the study protocol will be reported to the Research Ethics Committee.

#### Harms

Since there is no actual physical intervention applied to the participants, no harms are foreseen for this trial due to the nature of the intervention being psychoeducation training. However, the research team acknowledges that the contents of the intervention programme may evoke some discomfort associated with experience of stress and burnout. The contents of the intervention programme will cover ways to handle burnout and a stress reduction technique that could possibly alleviate the discomfort. Moreover, supporting professional services from the MHAHK will also be available and the research team can refer any participants requiring counselling and/or psychological intervention to the appropriate support services.

## Discussion

This trial aims to evaluate the efficacy of a workplace mental well-being intervention programme that involves both the organisation and the individual components in meeting the mental health needs of workers. This intervention is a novel approach to occupational health in an area which is underserved by research and which is receiving increasing attention. An adequately powered RCT is required to confirm that this dual approach of an organisation environmental scan and evidence-based psychoeducation training in mental health literacy is efficacious in increasing the MHL, reducing burnout and stress, as well as enhancing the health-related quality of life of the participants. The proposed trial method, a wait-listed cluster randomised control trial, is considered appropriate in terms of the study design in providing adequate strength of evidence as well as the ethical consideration that the controls should also gain the benefits of the intervention.

If the trial results are in line with the hypothesis that supports the efficacy of the intervention programme, this will provide an evidence-based approach for an effective workplace mental well-being intervention programme that could not only enhance the understanding of mental health issues, but also reduce work-related burnout and stress as well as increase workers’ quality of life. This intervention is scalable as it adopts the e-Health and e-Learning approach and is formulated as blended training consisting of a series of self-paced online e-Learning modules. This will provide a flexible platform for all workplaces and workers can attempt these modules at their own convenience and at their own pace. Companies can be encouraged to incorporate the intervention programme into the staff development programme as well as the staff Occupational Health and Safety framework. Regular training can be provided by MHAHK to the staff body as well as the unit management through an agreeable partnership.

## Trial status

- Protocol version 2.0_2018.12.14
- Recruitment commencement date: 1 January 2020
- Approximate recruitment completion date: 20 October 2020
- Refer to Additional file 3 for items from the World Health Organization Trial Registration Data Set

## Supplementary information

**Additional file 1.** SPIRIT 2013 Checklist: Recommended items to address in a clinical trial protocol and related documents.

**Additional file 2.** Participant Information and Consent Form.

**Additional file 3.** Items from the World Health Organization Trial Registration Data Set.

## Data Availability

The datasets used and/or analysed during the current study are available from the corresponding author on reasonable request.

## Abbreviations

ACC: Australia China Council
DASS: Depression, Anxiety, and Stress Scale
GLLAMM: Generalised linear latent and mixed model
HKMMS: Hong Kong Mental Morbidity Survey
HRQoL: Health-related quality of life
MBI: Maslach Burnout Inventory
MHAHK: Mental Health Association Hong Kong
MHFA: Mental Health First Aid
MHL: Mental health literacy
OECD: Organisation for Economic Co-operation and Development
PMC: Project Management Committee
SPIRIT: Standard Protocol Items: Recommendations for Interventional Trials
TWC: Tung Wah College
WES: Work Environment Scale
WHO: World Health Organisation
YLD: Years lost due to disability
