# Supplementary material for: Protocol for a phase III wait-listed cluster randomised controlled trial of an intervention for mental well-being through enhancing mental health literacy and improving work friendliness in Hong Kong
Source: Trials. 2019 Dec 4;20:672. doi: 10.1186/s13063-019-3748-y (PMC6894236; doi:10.1186/s13063-019-3748-y)
Supplement: Supplementary file 2 — Additional file 2. Participant Information and Consent Form. [file 13063_2019_3748_MOESM2_ESM.docx]

**Appendix A– Participant Information and Consent Form**


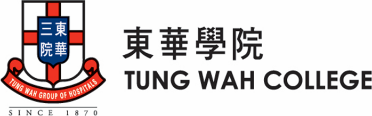

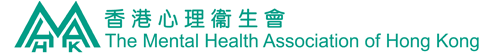


## INFORMATION SHEET

**Mental Wellbeing through Enhancing Mental Health Literacy and Improving Work Friendliness in Hong Kong: The WPMHL Project**

You are invited to participate in a joint project between the Tung Wah College (TWC) and Mental Health Association Hong Kong (MHAHK) supervised by Prof. Lawrence T. Lam and conducted by Professors. Lawrence T. Lam and his team.

There are two main aims of the study: 1) To implement and evaluate the Workplace Mental Wellbeing intervention program that responses to the organisation-and-individual Mental Health needs based on a dual approach. The program consists of an organisation environment scan and an evidence-based psychoeducation training in mental health literacy; and 2) To obtain information on the implementation of the program in order to maximise the scalability and transferability of the program to all workplace settings in Hong Kong and beyond. In the study, a workplace environment scan**,** using a standard protocol, will be conducted by a social worker with expertise in workplace issues. Information will be gathered via a staff survey and some face-to-face interviews to gain a better insight into the local issues of each site. De-identified and aggregated information collected from the assessment will be provided to the management of each participating site with professional interpretation of the findings and possible strategies for resolving the identified issues will be offered. The survey is not a test or an examination and there will not be any time limit for completion. There are also no risks involved in the study.

After the initial scan, you may also be invited to participate in a psychoeducation training program in Workplace Mental Health First Aid with stress and burnout prevention. The program will be conducted in both online and face-to-face modes with participants completing the online module and followed with a few hours of face-to-face contacts with the instructor. On the completion of the program and passing of a simple quiz, you will be recognized as a qualified Mental Health First Aider.

You have every right to withdraw from the study before or during the process without penalty of any kind. All information related to you will remain confidential unless it is summoned by the court. Information provided will be identifiable by codes known only to the researcher.

If you have any complaints about the conduct of this research study, please do not hesitate to contact Ms. Janet Yum, Secretary of the Research Ethics Committee of The Tung Wah College in person or in writing (c/o Research Office, Tung Wah College, 31 Wylie Road, Homantin, Hong Kong).

If you would like to obtain more information about this study, please contact Prof. Lawrence T. Lam at telephone number +852 3468 6805 or Ms Prudence Wong of the Mental Health Association Hong Kong at +852 2528 0196.

Thank you for your interest in participating in this study.

Prof. Lawrence T. Lam

Principal Investigator


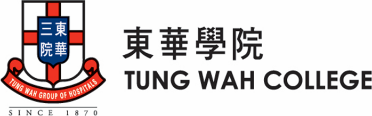

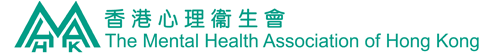


## CONSENT TO PARTICIPATE IN RESEARCH

**Mental Wellbeing through Enhancing Mental Health Literacy and Improving Work Friendliness in Hong Kong: The WPMHL Project**

I consent to participate in the captioned research supervised by Prof. Lawrence T. Lam and conducted by Professors. Lawrence T. Lam and his team.

The procedures as set out in the information sheet have been fully explained. I understand the benefits and risks involved. My participation in the project is voluntary.

I understand that information obtained from this research may be used in future research and may be published. I also understand that my right to privacy and anonymity will be protected, i.e., my personal details will not be revealed to any external parties and the information I provide on my workplace will not be revealed to my company.

I acknowledge that I have the right to question any part of the procedure and can withdraw at any time without penalty of any kind.

| ­­­­______________________ | ____________________ | ____________________ |
| --- | --- | --- |
| Signature of participant | Please PRINT name | Date |
| ­­­­______________________ | ____________________ | ____________________ |
| Signature of witness | Please PRINT name | Date |
| ­­­­______________________ | ____________________ | ____________________ |
| Signature of investigator | Please PRINT name | Date |


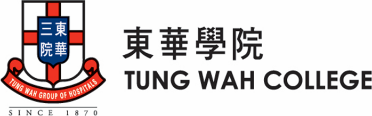

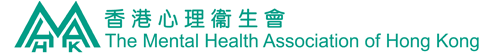


**WITHDRAWAL OF CONSENT**

**Mental Wellbeing through Enhancing Mental Health Literacy and Improving Work Friendliness in Hong Kong: The WPMHL Project**

I hereby wish to WITHDRAW my consent to participate in the study described above and understand that such withdrawal WILL NOT jeopardise my relationship with the ___________________________________[company name and the branch office].

**___________________________ _______________________ ____________________**

**Signature of participant Please PRINT name Date**

The section for Revocation of Consent should be forwarded to:

Professor Lawrence T Lam

Vice President (Academic)

Tung Wah College

31 Wylie Road, Homantin, Kowloon

Hong King SAR
