# Supplementary material for: Protocol for a phase III wait-listed cluster randomised controlled trial of an intervention for mental well-being through enhancing mental health literacy and improving work friendliness in Hong Kong
Source: Trials. 2019 Dec 4;20:672. doi: 10.1186/s13063-019-3748-y (PMC6894236; doi:10.1186/s13063-019-3748-y)
Supplement: Supplementary file 3 — Additional file 3. Items from the World Health Organization Trial Registration Data Set. [file 13063_2019_3748_MOESM3_ESM.docx]

| **Appendix B: Items from the World Health Organization Trial Registration Data Set** | |
| --- | --- |
| **Data category** | **Information** |
| Primary registry and trial identifying number | Australian New Zealand Clinical Trials Registry (ANZCTR): ACTRN12619000464167 |
| Date of registration in primary registry | 20/03/2019 |
| Secondary identifying numbers | N/A |
| Source(s) of monetary or material support | Health Care Promotion Scheme, Health and Medical Research Fund, Food and Health Bureau, Hong Kong Government. (Grant #02181028) |
| Primary sponsor | Tung Wah College, Hong Kong |
| Secondary sponsor(s) | Nil |
| Contact for public queries | ro@twc.edu.hk |
| Contact for scientific queries | Lawrencelam@twc.edu.hk |
| Public title | The Workplace Mental Health Intervention program for the enhancement of Mental Health Literacy and the reduction of stress and burnout among workers: The (WPMHL) Project |
| Scientific title | A phase III wait-listed RCT of a Workplace Mental Health Intervention program for the enhancement of Mental Health Literacy and the reduction of stress and burnout among workers |
| Countries of recruitment | Hong Kong |
| Health condition(s) or problem(s) studied | Mental Health, Stress, Burnout, Health-related Quality of Life |
| Intervention(s) | Environment and Individual-focused program with an environment scan and online psychoeducation training for workers |
| Key inclusion and exclusion criteria | Ages eligible for study: ≥18 years  Sexes eligible for study: both Accepts healthy volunteers: yes |
|  | Inclusion criteria: All staff members working at the selected sites are eligible to participate in the study. Participants must be willing to give written informed consent, and willing to participate to and comply with the study. |
|  | Exclusion criteria: Any staff member at any site who have already received a similar psychoeducation training program or involved in any similar intervention program before will be excluded from the current intervention program. |
| Study type | Wait listed cluster randomised controlled trial |
|  | Allocation: Using sites as the primary unit, simple randomisation using a randomisation table created by computer software |
|  | Primary purpose: Educational / counselling / training |
|  | Phase III |
| Date of first enrolment | Proposed September 1, 2019 |
| Target sample size | 400 |
| Recruitment status | Recruitment not yet commenced |
| Primary outcome(s) | Mean change in participants’ Mental Health Literacy scores. |
| Key secondary outcomes | Burnout, Stress, and Health-related Quality of Life |
